# Advancements in the application of brain-computer interfaces based on different paradigms in amyotrophic lateral sclerosis

**DOI:** 10.3389/fnins.2025.1658315

**Published:** 2025-12-08

**Authors:** Tong Li, Yuling Gao, Jiaqi Zhou, Yize Chen, Shengchao Zhang, Xiaoyang Gong, Yong Liu

**Affiliations:** Department of Rehabilitation Medicine, The First Affiliated Hospital of Dalian Medical University, College of Health-Preservation and Wellness, Dalian Medical University, Dalian, China

**Keywords:** amyotrophic lateral sclerosis, brain-computer interfaces, paradigms, neurodegenerative disease, advancements

## Abstract

Amyotrophic lateral sclerosis (ALS) is a progressive neurological condition that leads to the gradual loss of movement and communicative abilities, significantly diminishing the quality of life for affected individuals. Recent advancements in neuroscience and engineering have propelled the swift evolution of brain-computer interfaces (BCIs), which are now extensively utilised in medical rehabilitation, military applications, assistive technologies, and various other domains. As a communication medium facilitating direct interaction between the brain and the external world independent of the peripheral nervous system, BCI provides ALS patients with an innovative method for communication and control, offering unparalleled prospects for improving their quality of life. Recent collaborative endeavours among several specialists have markedly enhanced the precision and velocity of diverse BCI paradigms, signifying a breakthrough in BCI applications for ALS. Nonetheless, obstacles and constraints remain. This study methodically extracted pertinent literature from the Web of Science and PubMed databases in accordance with PRISMA guidelines. Following stringent inclusion and exclusion criteria, 23 studies were identified. This data allows us to summarise the application results and current limitations of several BCI paradigms in motor control and communication, while delineating prospects in multimodal fusion and adaptive calibration. This review presents evidence-based references for the effective translation and application of BCI technology in ALS rehabilitation.

## 1 Overview of ALS

ALS is a rare, lethal, progressive neurological condition, with a global prevalence of 1.68 per 100,000 individuals, exhibiting an increasing tendency annually (Feldman et al., 2022; Ilieva et al., 2023). ALS predominantly impacts motor neurons in the brain, brainstem, and spinal cord. Initial symptoms generally encompass limb weakness, dysphagia, and dysarthria, advancing to muscle weakness, atrophy, bulbar palsy, and pyramidal tract manifestations. Mortality frequently arises from respiratory failure (Feldman et al., 2022). As the condition advances, patients progressively lose motor function, resulting in restrictions in activities of daily living (ADL) and the need for long-term care and medical assistance. This extended caregiving not only places a significant financial strain on families but may also induce psychological stress and diminish the quality of life among family members (Xu et al., 2020). Moreover, ALS patients often encounter social isolation and psychological challenges, including despair and anxiety (Yu et al., 2024), Quantitative research on social isolation and psychological issues in ALS patients indicates that 38% of individuals experience considerable loneliness; a 1-point rise in loneliness scores is associated with a 0.42-point increase in depression symptoms, a 0.39-point increase in anxiety (both *p* < 0.001), and a 4.6-point reduction in quality of life scores. The overall disease burden in patients with concurrent loneliness and depression was 26% greater than in those without loneliness (Yu et al., 2024). Literature indicates that the prevalence of depression in ALS patients varies from 8 to 56%, with a median of 31%; the prevalence of anxiety is 46%, both significantly exceeding age-matched controls by 2–3 times (Yu et al., 2024).

Despite much research focused on its pathophysiology and therapeutic approaches, the precise aetiology remains inadequately elucidated. Studies suggest that genetic and environmental variables may interact to facilitate the emergence of ALS (Vasta et al., 2022). Genetic factors significantly influence the start of ALS, with around 10% of individuals possessing a familial history of the condition. Furthermore, extended exposure to environmental elements such as heavy metals, pesticides, and electromagnetic fields may elevate the risk of getting ALS. Smoking, heavy metal exposure, and pesticide exposure are correlated with increased risks of 38, 58, and 46%, respectively (Oh et al., 2025).

In this context, BCI technology is especially important for ALS patients, providing a non-muscular method of communication and control that allows them to engage with the external environment despite the loss of motor function (Angrick et al., 2023). BCI systems operate external devices, including wheelchairs, prostheses, and computers, by directly interpreting brain impulses, thereby augmenting patients’ self-care capabilities and autonomy (Kim H, et al., 2025). BCI technology can be utilised in rehabilitation training, employing neurofeedback to assist patients in recovering partial motor function and improving brain plasticity (Pirasteh et al., 2024). This technology enhances patients’ physical health while also mitigating psychological and social pressures, therefore improving their quality of life.

## 2 Overview of BCIs

BCI is a communication conduit that facilitates direct connection between the brain and external equipment, independent of the peripheral nervous system (Gao et al., 2021). By capturing brain signals produced during neural responses via invasive or non-invasive techniques, BCI translates these signals into diverse control signals or commands. This enables individuals to engage with their surroundings without dependence on verbal communication or physical actions, offering considerable potential for enhancing the quality of life for ALS patients (Hossain et al., 2022).

### 2.1 Classification and associated paradigms of BCIs

**Figure 1:**
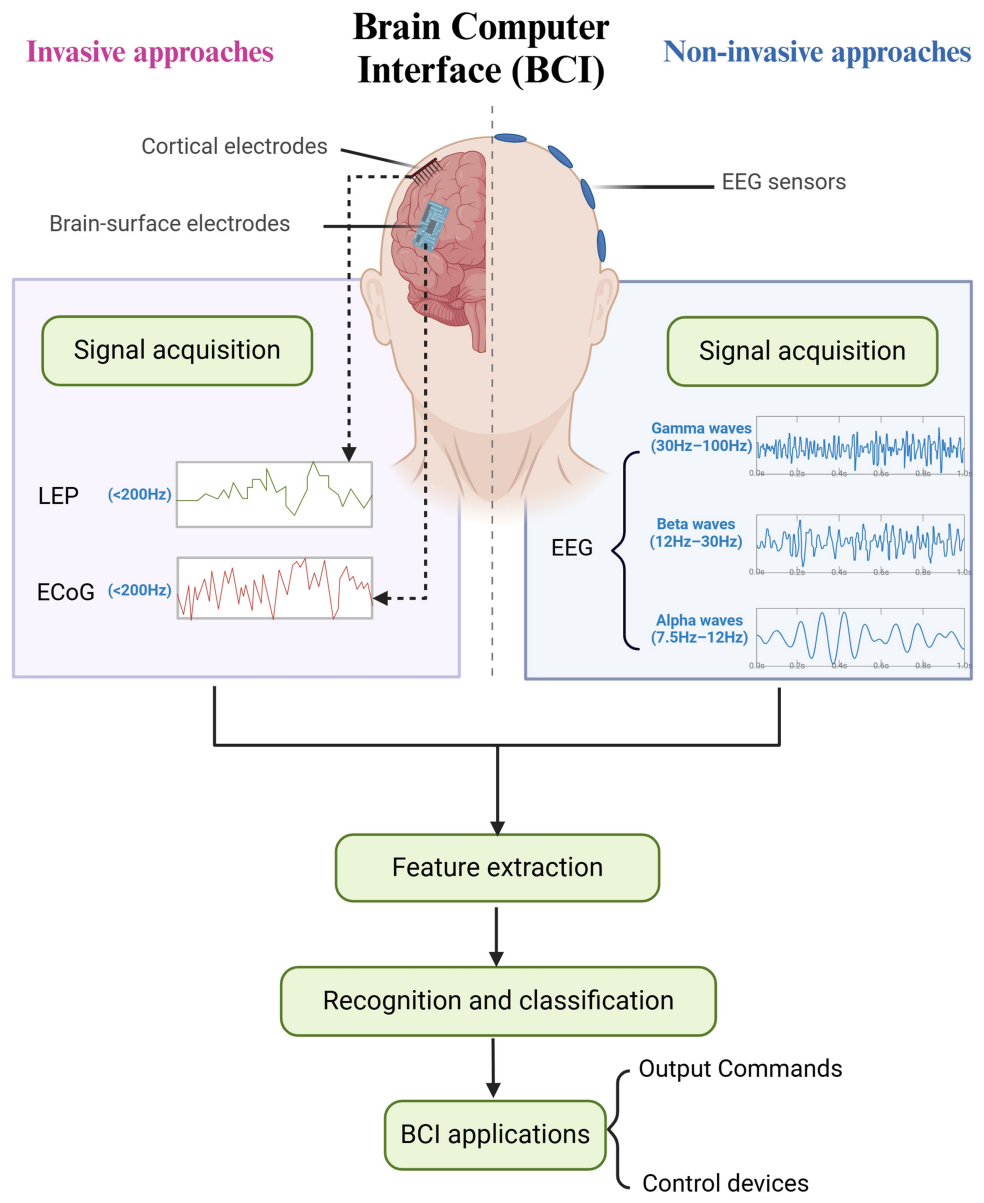
Brain-computer interface paradigms and mechanisms.

BCIs can be classified into invasive, semi-invasive, and non-invasive categories based on the extent of BCI implantation during signal acquisition. Each category includes several unique paradigms, each with specific advantages and disadvantages, rendering them appropriate for users with diverse requirements (Figure 1).

#### 2.1.1 Invasive BCIs

Invasive BCI is a technology that involves the surgical implantation of electrodes into the cerebral cortex or deep brain structures to record and decipher neural signals (Zhao et al., 2023). Invasive procedures are predominantly employed in urgent medical situations, such as the implantation of electrodes in paralysed patients to manipulate robotic limbs or facilitating text input for patients with LIS through cognitive processes. Currently, the invasive BCI primarily used in ALS is the intracortical BCI (iBCI; Peksa and Mamchur, 2023). iBCIs capture cerebral signals via deep electrodes. Microelectrode arrays are inserted into the cerebral cortex to record signals from deep neurones (Chaudhary et al., 2022). This method produces superior signals and facilitates the focused capture of particular types of cerebral electrical activity. Nonetheless, it entails comparatively elevated surgical risks, frequent problems, and necessitates prolonged maintenance following implantation (Hettick et al., 2025; Table 1).

**Table 1:** Pros and cons of invasive BCIs.

Paradigm	Pros	Cons
iBCIs	High information resolution, obtaining very high-precision signals; Capture signal details and record different types of neural signals in a targeted manner.	Highly invasive and risky surgery; Risk of causing an immune response in the body, poor biological compatibility, and affecting signal quality; Less stable, requiring frequency of calibration.

Recent years have shown significant progress in invasive BCI technologies. Companies such as Neuralink exemplify the new generation of invasive BCIs that utilise flexible electrode cables with diameters of merely a few micrometres. These cables are meticulously embedded in brain tissue by robotic technologies, facilitating wireless transfer of high-throughput data. An integrated ASIC processor processes and wirelessly transmits extensive brain data. This chip can link to a maximum of 96 “wires,” yielding a total of 3,072 channels. Each channel may sample at a frequency of 20,000 hertz, facilitating high-fidelity signal acquisition (Musk, 2019). The NeuroPort array has 96 stiff silicon microneedles designed to penetrate the cerebral cortex for stable recording of high-quality single-neuron activity signals. It contains comprehensive validated evidence on long-term implant stability and reliability, with patients utilising it regularly for over 8 years without reporting any major device-related adverse events (Handelman et al., 2022).

#### 2.1.2 Semi-invasive BCIs

Semi-invasive BCI holds an intermediary position between invasive and non-invasive BCI. Electrodes are generally positioned beneath the skull on the dura mater (or brain surface) during minimally invasive surgery, avoiding penetration of brain tissue. This methodology includes multiple modalities, including electrocorticogram-based BCI (ECoG-based BCI) and intravascular BCI. In contrast to non-invasive systems, semi-invasive electrodes are situated nearer to the cerebral cortex, facilitating superior signal collection and less cranial bone attenuation. They provide enhanced signal strength and resolution compared to non-invasive options. By circumventing direct penetration into brain tissue, they reduce the dangers of tissue damage and infection linked to completely invasive methods (Table 2).

**Table 2:** Pros and cons of semi invasive BCIs.

Paradigm	Pros	Cons
ECoG-based BCIs	Less invasive and less damaging to brain tissue; Acquire larger areas of brain signals; Good stability and no need for frequent calibrations.	Low signal resolution and poor signal quality.
Intravascular BCIs	Avoid craniotomy and reduce trauma; High signal-to-noise ratio; Signal stability.	The signal recording range is limited; Difficult to replace after long-term implantation.

##### 2.1.2.1 ECoG-based BCIs

ECoG is a neural interface consisting of an array of electrodes intended to capture neural activity on the brain’s surface. Usually positioned on the cerebral cortex surface or subdurally, it facilitates high-resolution recording of electrophysiological data (Chiang et al., 2020), rendering it one of the most prevalent semi-invasive BCI paradigms.

The insertion of traditional ECoG electrode arrays is a very intrusive surgical procedure (Coles et al., 2024) that may result in postoperative infections and other problems. Recent breakthroughs in materials science and micro/nanofabrication technologies have led to substantial progress in the design and production of ECoG electrodes. For example, a study (Coles et al., 2024) created an innovative ECoG electrode constructed from lightweight materials that can be folded in an origami manner. This electrode is inserted into the brain via a small drilled aperture in the skull, presenting a minimally invasive method that facilitates the therapeutic utilisation of extensive neural interfaces. Moreover, these novel ECoG electrodes exhibit superior performance in signal quality, biocompatibility, and long-term stability. Research has shown that flexible ECoG electrodes adhere better to the cerebral cortex, minimise signal interference, and have superior biocompatibility, facilitating prolonged implantation without considerable immune responses (Ji et al., 2023; Zhou et al., 2025). This not only improves ECoG-based BCI efficacy but also diminishes surgical risks and the complexity of postoperative care for patients. Nonetheless, the use of ECoG-based BCI in ALS patients continues to encounter numerous obstacles. The surgical implantation procedure necessitates highly specialised medical personnel and sophisticated equipment. Furthermore, prolonged implantation may result in electrode deterioration and possible injury to brain tissue (Yan et al., 2023). Consequently, subsequent investigations must enhance the design and fabrication processes of ECoG electrodes while advancing safer, more efficient, and dependable implantation methodologies.

##### 2.1.2.2 Intravascular BCIs

Intravascular BCI signifies a novel advancement in BCIs, functioning by accurately recording and interpreting neural activity within the brain (Wang et al., 2024). Neurons convey information via electrical signals that encapsulate extensive brain activity data, encompassing both basic sensory perception and intricate cognitive decision-making. Intravascular BCI focuses on acquiring cerebral electrical impulses to create a communication link between the brain and external devices (Soldozy et al., 2020). This approach entails the insertion of electrode scaffolds into principal brain vessels, such as the superior sagittal sinus, via endovascular techniques to monitor neural activity. It amalgamates the benefits of less invasive methodologies with superior signal quality, circumventing the hazards linked to craniotomy (Opie et al., 2018; Mitchell et al., 2023). Current clinical trial findings indicate its exceptional safety and efficacy, providing renewed optimism for the restoration of motor function in paralysed individuals. In recent years, firms have created the Stentrode system, comprising a mesh scaffold constructed from nickel-titanium alloy, embedded with microscopic electrodes that can record neural signals from surrounding brain tissue.

This technique, with its electrodes situated near brain neurones, can get better and more precise neural data. The signal quality markedly exceeds that of non-invasive brain electrodes, offering more dependable data support for the actual implementation of BCI technology. It exhibits distinctive application value in domains such as medical rehabilitation and brain research.

#### 2.1.3 Non-invasive BCIs

Non-invasive BCI does not necessitate surgical implantation; it instead acquires neural activity signals from the brain through external devices. Non-invasive BCIs categorised by signal acquisition method, encompass electroencephalography-based BCIs (EEG-based BCI), magnetoencephalography-based BCIs, and functional near-infrared spectroscopy-based BCIs (fNIRS-based BCI; Yang et al., 2021), with EEG-based BCI and fNIRS-based BCI being the most prevalent (Table 3).

**Table 3:** Pros and cons of non-invasive BCIs.

Paradigm		Pros	Cons
EEG-based BCIs	P300-based BCIs	Stable signal and high accuracy; Do not need complex training; Multi-target selection	Limited transmission rate; Variability across individuals.
	SSVEP-based BCIs	High transmission rate and speed; Less affected by individual differences.	Easy to cause visual fatigue of users; Signal easily interfered.
	MI-based BCIs	No need for external stimulation.	High training requirements; Limited applicability.
fNRIS-based BCIs		Insensitive to motion artefacts; Higher spatial resolution.	Slow transmission speed; Signal easily interfered.

##### 2.1.3.1 EEG-based BCIs

EEG-based BCI possesses a wide array of applications and provides benefits, including affordability and portability. Nevertheless, research demonstrates that EEG-based BCI is vulnerable to disruption (Meng et al., 2023). EEG-based BCI includes three primary paradigms: BCI utilising P300 event-related potentials (P300-based BCI), BCI employing motor imagery (MI-based BCI), and BCI based on steady-state visual evoked potentials (SSVEP-based BCI; Abiri et al., 2019).

P300 is an attention-associated event-related potential (ERP) that reflects the brain’s electrical activity in reaction to particular events or stimuli. P300-based BCI necessitates that participants concentrate on particular stimuli and react appropriately. About 300 milliseconds following the brain’s reception of the target stimulus, a positive deflection potential arises, representing the P300 ERP (Allison et al., 2020). The P300-based BCI is the most prevalent EEG-based BCI paradigm, distinguished by an extensive research history, advanced signal processing techniques, and remarkable stability.

Synchronised alterations in mu and beta rhythms transpire within the sensorimotor areas of the left and right cerebral cortex during physical movement, mental imagery, or sensory input. This results in either increased or decreased power in the EEG spectrum, specifically classified as Event-Related Desynchronization (ERD) and Event-Related Synchronisation (ERS; Xu et al., 2021). According to this theory, when individuals envision the movement of a particular body part, the associated motor cortex regions become activated. EEG apparatus captures the resultant cerebral impulses, which are subsequently interpreted by classification algorithms. This procedure correlates the user’s envisioned movement with external equipment (Wu, D. et al., 2022), forming the operational mechanism for MI-based BCI. This paradigm necessitates no physical movement from the patient; simply envisioning the movement captures the fluctuating EEG signals and obviates the requirement for eye-tracking.

When individuals are presented with visual stimuli of a specific frequency, consistent rhythmic brain signals associated with that frequency manifest in designated areas of the brain (Guney et al., 2022). These neural signals comprise SSVEP. SSVEP-based BCI provides an elevated signal-to-noise ratio (Chuang et al., 2022) and necessitates minimal user training (Guo et al., 2022). Nevertheless, SSVEP-based BCI necessitates that participants maintain fixation on flickering or moving visual stimuli for prolonged durations, which may readily induce visual fatigue (Guney et al., 2022). Moreover, SSVEP signals are consistently intertwined with typical brain activity and ambient noise, resulting in diminished signal quality (Tian et al., 2023).

##### 2.1.3.2 fNIRS-based BCIs

fNIRS is a neuroimaging modality appropriate for BCI that detects regions of brain activation by connecting neurophysiological activity with local hemodynamic alterations, encompassing fluctuations in oxygenated and deoxygenated haemoglobin levels (Li et al., 2025). By accurately quantifying fluctuations in haemoglobin levels, fNIRS adeptly identifies regions of brain activation, thereby creating a comprehensive “map” of cerebral activity. Near-infrared light encompasses wavelengths ranging from approximately 700 to 2,500 nm. In terms of scattering characteristics, near-infrared light exhibits reduced scattering relative to other light wavelengths while traversing biological tissue (Lu et al., 2024). It preserves directional stability more effectively during propagation, diminishing chaotic scattering and facilitating enhanced penetration into deeper tissue layers. Near-infrared light demonstrates modest absorption by biological tissues, striking a balance that neither obstructs propagation nor fails to provide adequate tissue information. It can infiltrate tissues to significant depths (Jiang et al., 2022).

Furthermore, fNIRS-based BCI provides significant portability (Martini et al., 2020; Paulmurugan et al., 2021), liberating BCI from laboratory limitations and expanding its potential applications in everyday life. This portability obviates the necessity for substantial, stationary apparatus or intricate procedures, like as surgical implantation, hence markedly reducing barriers to utilisation and related dangers. Users can now get brain signals in a relaxed, natural state, significantly broadening potential application contexts. Nonetheless, fNIRS depends on hemodynamic responses, which are comparatively sluggish and may result in signal latencies. Furthermore, the restricted quantity of sensors employed results in fNIRS demonstrating diminished spatial resolution (Xu et al., 2021).

## 3 Mechanisms of BCI application to ALS

Applications of BCIs for patients with ALS can be chiefly classified into communication-oriented BCIs and motor-assistance BCIs. In contrast to motor-assistance BCI, communication-based BCI is appropriate for a wider spectrum of ALS patients. The utilisation of BCI in ALS patients primarily encompasses the production, recording, and decoding of brain signals, followed by BCI command output and functional implementation.

### 3.1 Generation of neural signals

Patients with ALS undergo degeneration of motor neurons, impacting motor, cognitive, and linguistic skills. Language impairments present in several forms, such as challenges with naming, semantic recall, auditory understanding, sentence comprehension, and repetition (Ceslis et al., 2020). In ALS patients, the effort to speak activates distinct brain signals in critical language areas, including the ventral sensorimotor cortex, dorsal laryngeal motor cortex, and Broca’s area 55b (Card et al., 2024). These signals mirror brain activity detected in healthy speakers during verbal communication, storing information pertinent to voice, intonation, and speech tempo (Willett et al., 2023). Communication-based BCIs can identify and interpret brain signals, providing ALS sufferers with an alternative communication method. Recent research demonstrates that AI technology may produce synthetic speech that closely mimics the natural voices of ALS patients, markedly enhancing speech intelligibility and clarity, hence improving their quality of life (Regondi et al., 2025). Likewise, when ALS patients endeavour to initiate movement, motor-associated cerebral regions (including the primary motor cortex and premotor areas) emit neural signals. Assistive motor-based BCIs can acquire neural signals from ALS patients, translate them into commands for controlling external devices, and thereby facilitate motor function for these individuals.

Recent investigations have revealed that late-stage ALS patients may develop locked-in syndrome (LIS) and Complete locked-in syndrome (CLIS). LIS is defined as an extreme disability state caused by ventricular brain injury (Babiloni et al., 2010). LIS denotes a condition in which patients maintain consciousness while exhibiting near-total paralysis of voluntary muscles, with the exception of vertical eye movements and blinking. Conversely, CLIS patients forfeit the capacity for vertical eye movements and blinking, leading to a total cessation of motor output and incapacitating them from communicating with the external environment in any manner (Zilio et al., 2023). In contrast to healthy persons, EEG data from patients with LIS and CLIS demonstrate hypersynchrony, characterised by an absence of high-frequency activity above 10 Hz, alongside predominant high-amplitude slow waves within the 3–6 Hz range, with this phenomenon being more evident in CLIS patients (Secco et al., 2021). At present, almost all EEG-based BCIs depend on the alpha band and higher frequency bands (Lazarou et al., 2018). As a result, current BCI systems may find it challenging to facilitate meaningful communication with CLIS patients. Additional research and development of innovative BCI technology specifically designed for the neuroelectric activity profiles of CLIS patients is necessary. Moreover, alterations in the electrical characteristics of impaired neurones in ALS patients may influence the feasibility of several BCI paradigms. The P300 wave is an ERP that signifies the brain’s reaction to particular stimuli (Pan et al., 2022). Its generation entails the aggregation of postsynaptic potentials from neurons throughout many brain regions, including the sensory cortex and parietal cortex. In ALS patients, neuronal axons demonstrate hyperexcitability, resulting in ion channel malfunction that hinders action potential production and conduction. This modifies the amplitude and latency of the P300 wave, hence influencing the precision and rapidity of intent identification in P300-based BCI for ALS patients.

Moreover, ALS is a severely debilitating neurodegenerative disorder marked by the aberrant activation of glial cells in the patient’s brain, encompassing astrocytes and microglia. These glial cells assume a crucial and intricate role in the nervous system, engaging in neuroinflammation while simultaneously safeguarding neurons (Calafatti et al., 2023). Cytokines secreted by glial cells, including interleukin-1β (IL-1β) and tumour necrosis factor-*α* (TNF-α), are crucial throughout the implementation of BCI across many paradigms. Under typical physiological conditions, the concentration and distribution of these cytokines are meticulously regulated to preserve the homeostasis of the neurological system. In the brains of ALS patients, aberrantly activated glial cells secrete excessive cytokines, disturbing the natural equilibrium and creating an inflammatory milieu (Calafatti et al., 2023). MI-based BCI recognises and decodes the unique signals from motor cortex neurones to ascertain the user’s motor intent (Padfield et al., 2019). Nonetheless, the glia-mediated inflammatory milieu significantly disrupts the firing patterns of motor cortex neurones (Hu et al., 2022). As a result, the quality of brain signals produced by patients via muscular imagery markedly declines, hindering the BCI system’s ability to effectively interpret these signals. This significantly undermines the efficacy of MI-based BCI and may also lead to operational challenges and delayed reactions during system utilisation, profoundly affecting patients’ quality of life and rehabilitation advancement.

### 3.2 Recording and decoding of neural signals

BCIs acquire cerebral electrical impulses produced by ALS patients using invasive, semi-invasive, and non-invasive techniques, thereafter undergoing decoding and analysis (Peksa and Mamchur, 2023). Every BCI paradigm possesses unique advantages and limits. Intracortical electrode recordings yield signals with elevated spatial resolution and signal-to-noise ratio, essential for accurate control and decoding of intricate activities. Nonetheless, these techniques necessitate surgical electrode implantation, which entails risks of infection and possible long-term cerebral tissue damage, in addition to intricate procedures and substantial expenses. Non-invasive techniques such as EEG and fNIRS eliminate surgical hazards, providing significant portability and user-friendliness, so rendering them appropriate for prolonged and routine use. However, EEG is characterised by very low spatial resolution and vulnerability to external interference, and fNIRS has a restricted temporal resolution that may not satisfy real-time application requirements. Semi-invasive BCIs amalgamate the benefits of both invasive and non-invasive technology. By positioning electrodes on the cerebral cortex or within major blood vessels, they provide high-quality signals while minimising surgical risks and the potential for long-term problems, so presenting ALS patients with a safer and more effective alternative.

In addition to these prevalent signal acquisition methods, current studies suggest that functional magnetic resonance imaging (fMRI) can work as a potent complement for BCIs. fMRI identifies active areas in the brain by detecting blood oxygenation level-dependent (BOLD) signals. This data is utilised to identify appropriate target sites for electrode placement, facilitating BCIs in capturing brain signals with more precision (Leinders et al., 2023) and offering a more thorough comprehension of the brain’s general functional condition. Increased neuronal activity in a given brain region leads to a commensurate boost in local blood flow to supply additional oxygen and nutrients (Wu, Y. et al., 2022). These variations in blood flow generate subtle modifications in the local magnetic field. fMRI identifies these variances to produce images of cerebral activity, assisting BCI in pinpointing functional regions. Recent investigations indicate that fMRI can facilitate continuous speech reconstruction and the direct decoding of intricate semantics (Tang et al., 2023). And for the first time, a study has successfully depicted the activation time sequence of multiple language related brain regions in language tasks using high-resolution fMRI and time shift model analysis (Maruyama, 2021). Variations in BOLD values indicate modifications in local blood flow, which are strongly associated with neural activity. Research indicates that BOLD has a positive association with high-frequency bands in ECoG and a negative correlation with low-frequency bands in ECoG signals. Consequently, BOLD signals may also function as an indirect measure of a subject’s capacity to modulate ECoG signals more efficiently (Leinders et al., 2023). Thus, fMRI serves as an efficient technique for evaluating ALS patients’ competence in utilising BCIs and determining BCI efficacy.

Both fMRI and the aforementioned fNIRS depend on hemodynamic changes to measure brain activity, but each provides unique benefits in spatial and temporal resolution. fMRI has great spatial resolution, facilitating accurate localisation of active brain regions (Shin et al., 2024), but fNIRS possesses enhanced temporal resolution (Klein, 2024), permitting rapid detection of fluctuations in brain activity. This multimodal integration provides a more thorough solution for BCI applications in ALS patients, potentially improving BCI performance and reliability.

### 3.3 Command output and function implementation

BCI technology functions by receiving and interpreting electrical signals from the brain, transforming them into control commands for the operation of devices or systems. In ALS patients, the utilisation of both communication-based and motor-based BCIs markedly enhances quality of life and somewhat mitigates disease progression.

#### 3.3.1 Preserve neuronal activity in the cerebral cortex

ALS causes progressive loss of motor neurons, which gradually deteriorates the brain’s control over muscles (Hardiman et al., 2011). Communication-based BCI allows patients to communicate via brain signals, like envisioning certain movements or focusing on target directives. This mechanism boosts neuronal activity in various brain regions, including language centres and the motor cortex. According to the neuroplasticity hypothesis, the brain can preserve or improve functions with continued usage. As a result, important brain regions may preserve neural activity through BCI involvement, delaying cortical degeneration caused by disuse (Kim M. S, et al., 2025). Although direct studies on ALS patients are sparse, research on healthy people shows that long-term BCI training can improve functional connectivity in the cerebral cortex.

#### 3.3.2 Inducing alterations in synaptic plasticity

In ALS patients, aberrant activation of glutamatergic signalling pathways is intimately linked to neurodegenerative alterations (Arnold et al., 2024). Research demonstrates that the expression and functionality of AMPA and NMDA receptors are markedly modified in the brains of patients with ALS. Dysfunction of NMDA receptors impairs calcium influx, resulting in neuronal injury. Additionally, ALS patients demonstrate increased expression of GluN2A receptors and decreased expression of GluN2B receptors (Tian et al., 2021). These modifications in receptor subtype expression may hinder synaptic plasticity, compromising proper neuronal communication.

BCI technology presents a prospective intervention for ALS patients by preserving brain plasticity via the modulation of glutamate receptor subtype expression and function. Using SSVEP-based BCI as an illustration, when visual cortical neurones consistently receive stimuli of a given frequency and participate in BCI intention expression, the strength of their synaptic connections may be augmented or diminished. This modification of synaptic plasticity enhances patients’ adaptation to the BCI system, hence enhancing reaction efficiency to stimuli and the precision of information transmission. The BCI system may enhance synaptic effectiveness by promoting the development of AMPA receptors on postsynaptic membranes through targeted visual inputs. It may concurrently alter the phosphorylation state of NMDA receptors, affecting their expression and activity on postsynaptic membranes, thus augmenting synaptic plasticity. Moreover, BCI technology has the potential to repair synaptic plasticity and enhance inter-neuronal communication by modulating the expression of GluN2A and GluN2B receptors (Yee et al., 2018). This modulation aids in reducing neurotoxicity and postponing the advancement of neurodegenerative disorders, while potentially decelerating disease progression by improving brain plasticity and maintaining partial neural function.

## 4 Method

This review was executed and documented in accordance with the Preferred Reporting Items for Systematic Reviews and Meta-Analyses (PRISMA) standards.

### 4.1 Criteria for selection and eligibility requirements

The literature review was performed separately by Tong Li, Yuling Gao, and Jiaqi Zhou. All differences were addressed by dialogue and agreement with two senior reviewers, Xiaoyang Gong and Yong Liu. Due to the swift advancement of BCI technologies in the last decade, earlier literature fails to represent contemporary perspectives. The literature search encompassed the timeframe from 2015 to 2025. Initially, titles and abstracts were assessed, followed by a comprehensive examination of potentially eligible papers.

Inclusion Criteria: (I) Studies involving adults (≥18 years) diagnosed with ALS at any stage; (II) Studies employing any form of BCI; (III) Randomised controlled trials or observational studies published in English.

Exclusion criteria: (I) Studies involving minors; (II) Studies that failed to provide motor or cognitive outcomes or included patients with conditions other than ALS; (III) Non-peer-reviewed literature, encompassing reviews, editorials, and abstracts; (IV) Publications in languages other than English.

### 4.2 Sources of information and search methodologies

A thorough examination of pertinent literature was performed utilising the Web of Science and PubMed internet databases to find potentially qualifying publications. The reference lists of the included papers were manually examined to locate other pertinent articles.

The systematic search occurred from September to November 2025. The subsequent search phrases were employed:

**Table:** 

Serial Number	Search query
#1	“Amyotrophic Lateral Sclerosis”[MeSH]
#2	“ALS”[tiab] OR “Gehrig* Disease”[tiab] OR “Lou Gehrig Disease”[tiab] OR “Motor Neuron Disease, Amyotrophic Lateral Sclerosis”[tiab] OR “Lou Gehrig* Disease”[tiab] OR “Disease, Lou-Gehrigs”[tiab] OR “Charcot Disease”[tiab] OR “Amyotrophic Lateral Sclerosis With Dementia”[tiab] OR “Dementia With Amyotrophic Lateral Sclerosis”[tiab] OR “Amyotrophic Lateral Sclerosis, Guam Form”[tiab] OR “Guam Disease”[tiab]
#3	#1 OR #2
#4	“Brain Computer Interfaces”[MeSH]
#5	“Interface*, Brain-Computer”[tiab] OR “Neural Interface Technology”[tiab] OR “Interface Technolog*, Neural”[tiab] OR “Neural Interface Technologies”[tiab] OR “Technolog*, Neural Interface”[tiab] OR “Brain-Computer Interface”[tiab] OR “Interface*, Brain-Machine”[tiab]
#6	#4 OR #5
#7	“adult”[MeSH]
#8	“adults”[tiab]
#9	#7 OR #8
#10	#3 OR #6 OR #9

**Figure 2:**
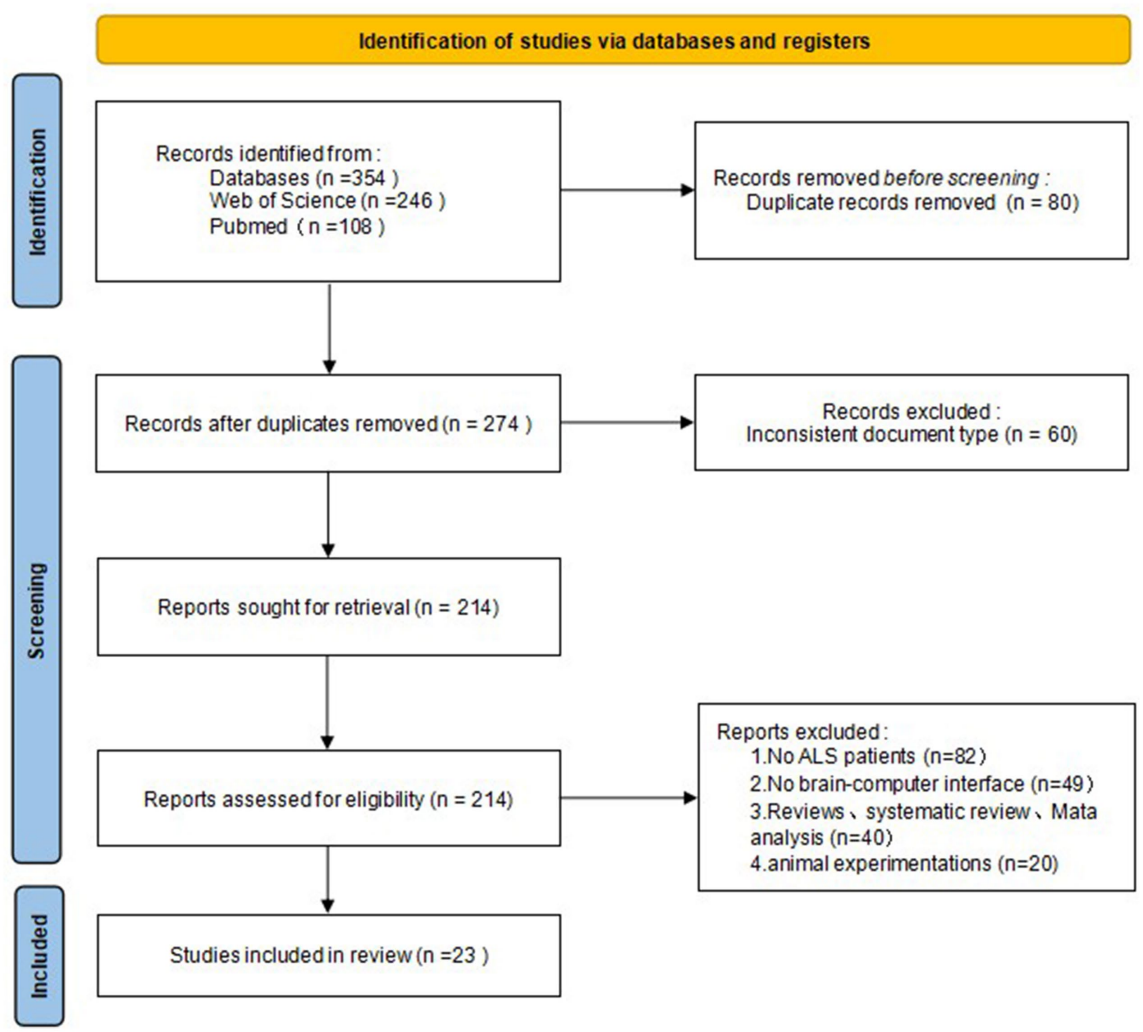
PRISMA workflow diagram for the included references.

A total of 354 records were identified: 246 from Web of Science and 108 from PubMed. After removing duplicates, 274 unique records remained. Of these, 23 articles (Vansteensel et al., 2016; Lim et al., 2017; Guy et al., 2018; Wolpaw et al., 2018; Hosni et al., 2019, 2020; Pels et al., 2019; Borgheai et al., 2020; Delijorge et al., 2020; Aliakbaryhosseinabadi et al., 2021; Oxley et al., 2021; Savic et al., 2021; Chaudhary et al., 2022; Chuang et al., 2022; Pires et al., 2022; Luo et al., 2023; Mitchell et al., 2023; Angrick et al., 2024, 2025; Card et al., 2024; Séguin et al., 2024; Wyse-Sookoo et al., 2024; Wairagkar et al., 2025) passed the initial title and abstract screening and were further assessed for eligibility through full-text review (Tables 4–6 and Figure 2).

**Table 4:** PICO for invasive BCIs.

Author-year	Population (P)	Intervention (I)	Comparison (C)	Outcome (O)	Study design (S)
Angrick et al. (2025)	62y male ALS patient (dysarthria)	Unsupervised speech activity detection via ECoG	Supervised VAD models (trained on acoustic speech)	Median alignment error (~530 ms) Real-time latency (10 ms) Speech detection rate (0.79) False alarm rate (0.10)	Prospective pilot study
Wyse-Sookoo et al. (2024)	61y male ALS patient (dysarthria)	Chronically implanted ECoG	Self-longitudinal comparison (12 months)	Signal stability Offline decoding accuracy (syllable/vowel/consonant) Electrode impedance	Longitudinal observational study (12-month follow-up)
Angrick et al. (2024)	60y male ALS patient (dysarthria)	Chronically implanted ECoG	Patient’s natural speech (6 keywords)	Synthetic speech intelligibility (80% human recognition) Spectral correlation (avg. 0.67) Alignment error (median 235 ms)	Prospective clinical pilot
Luo et al. (2023)	61-year-old male ALS patient (severe dysarthriay)	Chronically implanted ECoG	Self-functional comparison (3 months, different tasks)	Decoding accuracy (median 90.59%) Error rate (0.19 false/missed detections/min) System latency (1.24 s) Silent speech accuracy (median 85.18%)	Prospective intervention study (3-month real-time test)
Pels et al. (2019)	58-year-old female late-stage ALS patient(CLIS)	Fully implanted ECoG-BCI	Self-longitudinal comparison (36 months)	BCI control accuracy (avg. 91%) High-gamma signal stability Electrode impedance Home usage (max. 148 h/month)	Longitudinal follow-up study (36-month monitoring)
Mitchell et al. (2023)	4 males (severe bilateral upper-limb paralysis; 3 ALS, 1 primary lateral sclerosis)	Fully implanted endovascular BCI	Self-comparison over 12 months	No device-related serious AEs/vessel occlusion/migration Stable signal (233 ± 16 Hz bandwidth) Typing: 93.9% ± 1.8% accuracy, 16.6 ± 5.6 CCPM	Prospective first-in-human study
Oxley et al. (2021)	2 males with ALS	Minimally invasive endovascular BCI	Self-comparison	No device-related AEs/thrombosis/migration Typing: 92.63–93.18% accuracy, 13.81–20.10 CCPM	Prospective early feasibility study

**Table 5:** PICO for semi-invasive BCIs.

Author-year	Population (P)	Intervention (I)	Comparison (C)	Outcome (O)	Study design (S)
Wairagkar et al. (2025)	45y male ALS, (severe dysarthria limited)	256 microelectrodes (left ventral precentral gyrus)	Residual dysarthric speech, acausal synthesis	Intelligibility (94.34% transcript accuracy, 34% PER) Latency (≤10 ms) Paralinguistic modulation	Single-case, 489-day follow-up
Card et al. (2024)	45y male ALS, (tetraparesis + severe dysarthria)	256 microelectrodes (left ventral precentral gyrus)	Residual speech (caregiver interpretation), head mouse	Accuracy (99.6%/50-word,97.5%/125 k-word) Rate (32 words/min) 8.4-month stability	Single-case, 8.4-month follow-up
Chaudhary et al. (2022)	34y male ALS (CLIS)	2 × 64 microelectrodes (SMA + M1)	Failed non-invasive BCIs	Intelligibility (44/107 days) Rate (1.08 chars/min) 86.6% neurofeedback accuracy	Single-case, 462-day follow-up
Vansteensel et al. (2016)	58y female ALS(CLIS)	Fully implanted BCI (left motor cortex electrodes + chest transmitter)	Eye-tracking device	Spelling accuracy (89%) Rate (2 letters/min) Device satisfaction	Single-case, 266-day follow-up

**Table 6:** PICO for non-invasive BCIs.

Non-invasive BCIs	Author-Year	Population (P)	Intervention (I)	Comparison (C)	Outcome (O)	Study design (S)
FNIRS-BCI	Hosni et al. (2020)	8 ALS patients (including 1 with late-stage LIS)	Recorded fNIRS signals during motor imagery	Direct comparison with EEG-BCIs	Mean classification accuracy: 85.4 ± 9.8% F-score: 0.87 ± 0.09	Single-centre feasibility study
	Borgheai et al. (2020)	6 ALS patients (including 1 with late-stage LIS)	Implemented fNIRS-VM paradigm	Direct comparison with EEG-P3S	Mean accuracy of VM paradigm: 81.3 ± 5.7% (higher than P3S’s 74.0%) Longitudinal accuracy of LIS patient: 73.2% ± 2.0% (higher than P3S’s 61.8%)	Single-centre comparative study
MI-BCI	Savic et al. (2021)	8 healthy + 2 ALS patients	EEG (10-ch) + EMG; assistive glove; self-paced reach-grasp task	Internal comparison between NG vs. G blocks	Median TPR = 0.8, PPV = 0.77 (no NG-G difference) ALS TPR = 0.75–0.95, PPV = 0.68–0.95	Feasibility study
	Hosni et al. (2019)	11 healthy + 6 ALS patients	EEG (13-ch)	ALS patients vs. healthy	ALS: reduced μ/β ERD (RMI prep/imagine, *p* < 0.05) β-ERD correlates with worse bulbar/cognitive function	Cross-sectional study
	Aliakbaryhosseinabadi et al. (2021)	30 ALS patients	EEG recording for hand movements; individualised selection of channels, features	Unified optimal channel (Cz) + classifier (LSVM) for all patients (no individual tuning)	Classification accuracy, sensitivity, specificity; correlation with disease factors	Cross-sectional Study
P300-BCI	Guy et al. (2018)	20 ALS patients	43-key keyboard; optimal flash stop + word prediction	Copy vs. free spelling; with vs. without word prediction	1.100% task completion 65% free spelling accuracy >95%; ITR = 17.72 bit/min	Prospective clinical study
	Séguin et al. (2024)	18 healthy + 7 severe motor-disabled	Auditory BCI (EEG), “yes/no” speech stimuli	patients vs. healthy subjects	Healthy: 86% accuracy Patients: 2/7 (29%) achieved 100% (ALS), others near chance	Feasibility study
	Pires et al. (2022)	10 healthy + 1 ALS	2 P300-BCI paradigms, 3D spatial audio + 16-ch EEG	AU-S vs. HVA-S	Healthy: HVA-S (90.5%) > AU-S (71.3%) Patient: AU-S 30% accuracy, HVA-S at chance	Exploratory study
	Delijorge et al. (2020)	18 healthy + 8 ALS	P300-BCI + robotic hand orthosis, 6 movements, CCA + RLDA	With vs. without orthosis	Mean online accuracy 89.8%, ITR 18.1 bit/min;	Clinical validation
	Wolpaw et al. (2018)	42 advanced ALS	Home P300-BCI (speller/email), 8-ch EEG, quarterly support	Self-comparison across stages	33% completed study; mean accuracy 73%, daily use 1.3 h; benefit > burden	Longitudinal cohort
SSVEP-BCI	Chuang et al. (2022)	24 healthy	Single-channel SSVEP-BCI; MTL deep neural network	DAE alone; CCA method	MTL: SNR = 6.86 dB, accuracy = 93.44% (higher than DAE/CCA)	Feasibility study
	Lim et al. (2017)	14 healthy + 3 severe ALS	SSVEP brain switch; 3-channel EEG; Skype call trigge	ALS patients vs. healthy	Healthy: 9.9 s call time, 191.9 s idle; ALS: 6.56 s call time; stable 4w use	Clinical feasibility study

## 5 The application of invasive BCIs in ALS

In recent years, advancements in intracortical electrode recording paradigms have progressed swiftly due to the joint efforts of several researchers.

Vansteensel et al. (2016) developed the inaugural “percutaneous lead-free, fully implanted” subdural ECoG-based BCI, allowing a late-stage ALS patient to type at home by merely “thinking about moving their hand,” with an accuracy rate of 89%. Recently, Card et al. (2024) and Wairagkar et al. (2025) presented an innovative intracortical electrode recording paradigm for BCI-assisted communication in ALS patients. This BCI operates as an intracortical speech neural prosthesis, exhibiting superior decoding precision, communication velocity, and reliability. In addition to implanting electrodes in conventional speech-related regions, the study also focused on the language area 55b, thereby expanding the range of neural signal acquisition to encompass more extensive brain activity data (Card et al., 2024). This region is a distinct area in the brain related to speech production, situated in the ventral section of the anterior central gyrus and intricately connected to speech generation.

Considering that late-stage ALS patients endure CLIS and LIS, audio feedback may serve as a conduit for these individuals to interact with the external environment. Chaudhary et al. (2022) developed an auditory feedback-based BCI for patients with CLIS due to ALS. Patients acquire the ability to control their neuronal firing rate through real-time pitch feedback, aligning their neural activity with target pitches. This allows them to choose certain letters to construct words and phrases, facilitating genuinely “motionless” communication.

## 6 Application of semi-invasive BCIs in ALS

### 6.1 Application of semi-invasive BCIs in ALS

Recent research by specialists on ECoG-based BCI for ALS has concentrated on two primary aspects: (1) the stability and accuracy of ECoG-based BCI; (2) investigating methods to improve signal decoding speed.

The Wyse-Sookoo et al. (2024) executed a 12-month study with mid-stage ALS patients, evaluating the durability of ECoG signals, specifically the consistency of high-gamma signals for long-term speech BCI. Pels et al. (2019) executed a 36-month study with late-stage ALS patients to assess the stability of ECoG signals, particularly high-gamma signals, for long-term speech BCI applications. In Target tasks, users consistently exhibited good performance (average accuracy 91%, empirical chance level 48.4%, no significant trend), signifying steady BCI control signals. Subject satisfaction with the BCI device was notably elevated. Both investigations, however, possess limitations: each involved only one ALS patient and examined solely single-syllable letters, rendering them unable to assess the stability of ECoG signals in more intricate linguistic contexts.

Prior research and applications indicate that most ECoG-based BCI systems often necessitate regular calibration, demanding considerable time and effort from both patients and staff. Recently, specialists developed an ECoG-based BCI system that needed no recalibration (Luo et al., 2023), in stark contrast to other systems that required periodic recalibration to sustain performance. Patients attained a median accuracy rate of 90.59% (95% confidence interval: [89.47, 92.00%]), demonstrating consistent performance across the research duration. This illustrates that the gadget improves communication efficiency while thoroughly addressing patient experience and practical requirements. The introduction of this innovative ECoG-based BCI provides a more convenient, efficient, and user-friendly communication method for individuals with severe ALS. Moreover, Angrick et al. (2024) showcased an innovative online voice synthesis ECoG-based BCI that allows users to articulate thoughts in real-time via the BCI, hence enhancing timely communication while generating speech that retains the individual’s own vocal traits. A study (Angrick et al., 2025) accomplished the inaugural detection of speech activity utilising unlabeled ECoG data, so transcending the conventional BCI dependence on EEG-acoustic annotations and offering a framework for the unsupervised analysis of speech-based BCIs.

### 6.2 Application of intravascular BCIs in ALS

Intravascular electrode stents are a revolutionary semi-invasive BCI technique that has evolved recently. This method facilitates high-resolution recording of EEG signals while presenting a reduced wound surface relative to completely invasive BCIs. Clinical investigations have shown its advantageous safety profile and initial efficacy. A research (Mitchell et al., 2023) including five patients with profound bilateral upper limb paralysis was conducted over a 12-month follow-up period. The results indicated the absence of significant adverse events, vascular blockage, or device displacement. Subsequent imaging verified that the gadget was firmly situated within the blood vessel. Furthermore, a separate study (He et al., 2024) elucidated the vascular interventional procedure for the Stentrode system and presented preliminary clinical trial outcomes, affirming its elevated signal-to-noise ratio for neural signal acquisition and the lack of serious adverse events associated with the device during clinical trials. Additionally, a study conducted by Oxley et al. (2021) involved two ALS patients exhibiting upper limb flaccid paralysis, in which the innovative intravascular Stentrode BCI was implanted into the superior sagittal sinus using minimally invasive neurointerventional surgery. Postoperatively, patients engaged in machine learning-assisted training to regulate diverse mouse operations (e.g., zoom, left-click) by decoding wirelessly transmitted ECoG signals during attempted movements. This facilitated the operation of computer systems through the integration of eye-tracking for cursor navigation. Results indicated that both patients commenced unsupervised home utilisation of the device at 71 and 86 days following surgery, respectively. With predictive text deactivated, Patient 1 attained an average typing accuracy of 92.63% and a typing speed of 13.81 correct characters per minute (ccpm); Patient 2 earned an average accuracy of 93.18% and a typing speed of 20.10 ccpm. Significantly, both patients autonomously executed instrumental activities of daily living (IADLs), including texting, online purchasing, and financial management, thereby validating the technology’s efficacy in promoting functional independence.

## 7 Application of non-invasive BCIs in ALS

### 7.1 Application of P300-based BCIs in ALS

The P300-based BCI paradigm necessitates little motor abilities from patients, provides high precision, and accommodates several stimulus modalities, including visual and aural inputs. Guy et al. (2018) assessed the efficacy of P300-based BCI in facilitating daily communication for ALS patients. The study included 20 ALS patients, and the results indicated that all participants successfully accomplished spelling exercises. In the replication spelling challenge, all participants attained over 90% correct letter selection, with 60% achieving 100% accuracy. In free spelling exercises, 95% of participants attained above 75% accuracy in symbol selection, with 65% surpassing 95% accuracy. Delijorge et al. (2020) created a BCI system that employs P300 potentials to operate robotic hand orthoses, allowing ALS patients to exert mental control over hand movements. Experimental findings indicated that both healthy individuals and ALS patients displayed high categorisation accuracy and information transfer rates in both offline and online assessments. Wolpaw et al. (2018) examined the autonomous home utilisation of P300-based BCI by patients with ALS. Results demonstrated consistent system functioning over a period of up to 18 months during autonomous home utilisation by patients with late-stage ALS.

Prior P300-based BCI systems mostly employed visual stimuli. Recently, studies have concentrated on advancing P300-based BCI utilising aural stimuli or audiovisual integration. Nonetheless, application results have been suboptimal, and the technology persists in encountering various hurdles necessitating additional progress. Séguin et al. (2024) evaluated the efficacy of an auditory-based P300-based BCI in healthy individuals compared to patients with significant motor deficits. The results demonstrated inadequate control efficacy: of the 7 patients with significant motor impairments, only 2 ALS patients attained 100% online accuracy, while the other 5 patients were unable to control the BCI online, exhibiting accuracy rates at or below random levels. Pires et al. (2022) highlighted visual–auditory consistency by comparing pure auditory BCI with mixed audiovisual BCI for ALS patients. Results suggested that while CLIS patients exhibited some distinction between target and non-target responses, online BCI performance was subpar, with accuracy rates falling below the level necessary for effective communication.

### 7.2 Application of SSVEP-based BCIs in ALS

Conventional SSVEP-based BCIs mostly utilise black-and-white checkerboard visual stimuli to provoke SSVEP responses. These sensations are exceedingly powerful and necessitate users to sustain lengthy, intense concentration, frequently resulting in visual fatigue or headaches (Lim et al., 2017). Lim et al. (2017) pioneered the utilisation of SSVEP-based BCI employing colour visual stimuli for patients with ALS. These colour stimuli provide enhanced comfort, and the system sustained satisfactory performance for a minimum of 4 weeks without necessitating revisions to the original calibration data.

In actual applications, SSVEP signals are frequently compromised by external noise, significantly affecting the efficacy of SSVEP-based BCIs. Consequently, creating an SSVEP-based BCI that can function reliably in loud surroundings is essential for aiding ALS patients in external communication. Chuang et al. (2022) devised a single-channel deep neural network system utilising multi-task learning (MTL) for SSVEP-based BCI. This system utilises a cohesive signal processing method that closely interlinks and mutually improves denoising and classification functions. The research indicated that the MTL-based system markedly surpassed the non-MTL system (*p*-value = 0.02088, significance level *p* < 0.05). Consequently, MTL significantly improves classification efficacy while reducing noise interference.

### 7.3 Application of MI-based BCIs in ALS

MI-based BCIs primarily rely on ERD and ERS signals. In recent years, numerous specialists have expressed interest in the potential application of Movement-Related Cortical Potentials (MRCP) for MI-based BCIs. MRCP is a naturally occurring cerebral wave signal linked to both motor execution and imagery. Its prolonged length distinguishes MRCP and rather gradual alterations, setting it apart from ERD/ERS signals in terms of both temporal and spectral attributes. Savic et al. (2021) devised an MRCP-based MI BCI to facilitate grasping assistance for ALS patients who possess limited reaching and gripping capabilities in the first phases. Test results indicated that certain patients attained control levels similar to or nearing those of neurologically intact individuals. Two ALS patients exhibited exceptional BCI control, with one attaining a True Positive Rate (TPR) of 0.95 and a Positive Predictive Value (PPV) in the glove-wearing BCI evaluation domain. This device, specifically engineered for ALS sufferers, more effectively addresses their distinct requirements. Aliakbaryhosseinabadi et al. (2021) conducted a study involving 30 ALS patients at different phases of the disease, from mild disability to CLIS. Participants executed hand movement tasks or visualised hand movements as directed. Findings indicated that patients at various disease stages successfully differentiated between hand movement and rest periods with excellent accuracy, exhibiting an average motion detection accuracy of 80.5 ± 5.6%. Notwithstanding the differing severity of the condition, shown by an average ALSFRS-R score of 23.9 ± 14.8 (out of 48) and an average hand muscle strength score of 2.1 ± 1.3 (out of 5), the detection performance of the BCI system remained predominantly stable. Consequently, the MRCP-based MI-BCI method may be relevant for ALS patients at any phase of the disease.

The disparities in neural electrical signals between ALS patients and healthy individuals during motor imaging have substantial implications for the advancement and enhancement of MI-based BCI systems. Hosni et al. (2019) performed a comparison analysis between ALS patients and healthy subjects. Findings demonstrated that ALS patients typically displayed less ERD during motor imagery. Additionally, a delayed ERD was noted in the contralateral sensorimotor area during the mu band and in the ipsilateral premotor area during the beta band. These discrepancies indicate possible impairment in the cortical neural networks related to motor planning and imagery in ALS patients. Researchers are optimistic about developing more customised MI-based BCI systems specifically designed for ALS patients, based on their distinct neural signal patterns.

MI-based BCI generally operates as a motor BCI that augments or substitutes motor function in ALS patients, largely by acquiring electroencephalographic signals from the primary motor cortex (Aliakbaryhosseinabadi et al., 2021; Savic et al., 2021). The primary motor cortex is the principal area of the brain that governs voluntary movement, functioning as both the source and main executor of motor control. It is essential for motor learning and collaborative interaction with other brain regions (McColgan et al., 2020; Aliakbaryhosseinabadi et al., 2021). Nonetheless, contemporary motor BCI research mostly concentrates on stroke and spinal cord injury patients, with comparatively few investigations addressing ALS patients. Additional expert scrutiny and investigation are required in this domain. Future motor BCIs are expected to deliver highly individualised movement aid solutions tailored to the varied requirements of ALS patients.

### 7.4 Application of fNIRS-based BCIs in ALS

fNIRS is a method that employs the propagation properties of near-infrared light for accurate localisation of target objects. Haemoglobin in cerebral tissue is the principal absorber of near-infrared light, with oxyhemoglobin and deoxyhemoglobin displaying unique absorption characteristics at varying wavelengths of near-infrared light (Huo et al., 2024). Activation of neurones in particular brain regions initiates the local neurovascular coupling process, resulting in a functional hyperemia response. This results in a substantial augmentation in cerebral blood flow in that region, together with an elevation in oxygenated haemoglobin levels and a corresponding reduction in deoxygenated haemoglobin levels (Muñoz et al., 2023), producing distinctive hemodynamic alterations. The alteration in concentration directly affects the absorption of NIR light in tissue, producing discernible alterations in optical signals. This facilitates high spatiotemporal resolution localisation of functional brain activity regions (Lu et al., 2024), ensuring accurate neural activity localisation for BCI system placement and neural signal acquisition.

In investigations of BCI applications for ALS patients, fNIRS technology has shown considerable benefits. Borgheai et al. (2020) introduced a “visual-mental (VM)” fNIRS-based BCI designed exclusively for late-stage locked-in ALS patients. This VM-based fNIRS-based BCI integrates visual activities with cognitive (arithmetic) processes, emphasising mental tasks above visual capabilities. The research involved six ALS patients and evaluated the accuracy rates of P300-based BCI in comparison to fNIRS-based BCI. The findings revealed that the mean accuracy rate for fNIRS-based BCI was 81.3% ± 5.7%, but the P300-based BCI achieved a lower rate of 74.0% ± 8.9%. Consequently, fNIRS-based BCI may be more appropriate for application in advanced ALS patients. Hosni et al. (2020) examined the viability of fNIRS for MI-based BCI. Patients with ALS engaged in three mental motor tasks utilising visual stimuli: left-hand movement imagery, right-hand movement imagery, and a resting state. fNIRS identified alterations in blood oxygenation within the prefrontal cortex and primary motor cortex during movement imagery. The findings indicated an average categorisation accuracy of 85.4% ± 9.8%. Consequently, fNIRS-based MI-based BCI presents potential as an innovative device system providing communication and control functionalities for ALS patients.

## 8 The application of BCIs combined with other technologies in ALS

### 8.1 Combined with vibrotactile stimulation

The amalgamation of BCIs with vibrotactile stimulation presents ALS patients with an innovative rehabilitation and assistance methodology. This combination promotes patients’ perception and control capacities while simultaneously improving BCI system performance and user experience through multimodal feedback. Studies have shown that administering vibrotactile stimulation at varying frequencies to the index fingers of both hands enables patients to direct their attention to the target side according to interface cues. This facilitates the acquisition and analysis of EEG data to interpret human intent, translate it into specific commands, and enable interaction with the external environment (Hehenberger et al., 2020). This method does not depend on visual or auditory channels, rendering it suitable for ALS patients with compromised vision or hearing yet preserved somatosensory systems.

Research has facilitated ALS patients in manipulating the position of a virtual cursor through MI-based BCI. Besides visual display, cursor position is communicated to users via tactile feedback by varying the intensity of vibrotactile stimulation in the upper limbs (Miao et al., 2020). The results indicated that individuals were able to operate the BCI only through vibration feedback, attaining an average accuracy of 56% and a peak of 72%. This illustrates that vibration feedback is a potent biofeedback technique for controlling MI-based BCIs.

The amalgamation of BCI with vibrotactile stimulation provides ALS sufferers with a multifaceted practical remedy. By selectively interpreting patient intent via vibrotactile inputs, it facilitates effective communication with the external environment and interaction with surroundings. This method can help evaluate cognitive performance in people with severe disorders such as LIS. Moreover, vibrotactile feedback augments patients’ control over the BCI system, offering a novel and practical technical approach to enhance quality of life and increase interactive autonomy.

### 8.2 Combined with artificial intelligence

In recent years, the swift progression of artificial intelligence (AI) technology has markedly improved BCI technologies. Concerning signal decoding, AI systems can interpret brain signals obtained from BCIs and translate them into distinct actions or orders (Zhang et al., 2020). Kalani and Anjankar (2024) developed an AI model utilising Graph Attention Networks (GAN) to interpret brain data from ALS patients. This model enhanced the classification accuracy of motor intents to an average of 74.06% by concentrating on intricate communication patterns among various brain areas. Furthermore, Francis Willett’s team created an iBCI that instructs AI software to transcribe neural activity from the brains of ALS patients into text instantaneously. This device attains a typing pace of 62 words per minute, closely approximating that of healthy individuals (Willett et al., 2021).

Real-time feedback systems are another notable application of BCI coupled with AI. By creating low-latency, highly resilient real-time processing algorithms, BCI systems can swiftly analyse brain states and deliver feedback (Jin et al., 2024), thereby improving therapy efficacy and expediting patient recovery. Additionally, AI technologies can enhance the feedback mechanisms of BCI systems by utilising deep learning and analysing brain signals, resulting in increased personalisation and accuracy (Calderone et al., 2024).

### 8.3 Combined with smart wheelchair technology

Research demonstrates that MI-based BCI allows ALS patients to effectively control smart wheelchairs; nonetheless, these patients necessitate prolonged training durations and display comparatively low accuracy, highlighting the need for additional optimisation specific to their characteristics (Li et al., 2020). Chen et al. (2022) endeavoured to implement an SSVEP-based BCI system for electric wheelchairs, facilitating transitions between various scenarios. Patients with ALS attained accuracy rates of 80% across all scenarios, peaking at 90.7%, demonstrating the system’s responsiveness to diverse user requirements. This study was conducted exclusively with healthy persons, and its applicability and efficacy in the ALS patient population remain unverified.

The amalgamation of BCIs with intelligent wheelchair systems facilitates autonomous operation of wheelchairs, markedly improving mobility independence and self-care capacities for ALS patients. This enables individuals to engage more autonomously in social activities while diminishing dependence on others. Current research has insufficiently addressed the needs of ALS patients, necessitating researchers to enhance technology, broaden functionality, and elevate the user experience.

## 9 BCI adaptation for ALS patients at different stages and prospects for multi-technology integration

### 9.1 Advancement of ALS and BCI adjustment patterns

Patients with ALS demonstrate heterogeneity, since the sequences and presentations of disease progression differ among people (Ilieva et al., 2023). Diverse ALS patients possess unique requirements at different phases of the disease, and the appropriate forms of BCI vary accordingly. Nonetheless, no research has explicitly examined the correlation between BCI types and various illness stages in ALS patients. For early-stage ALS patients with partially preserved motor functions, non-invasive BCIs, such as MI-based BCIs, generally meet fundamental communication requirements. These devices provide intuitive operation and enhanced safety, allowing patients to retain communication abilities without extra physical strain, as evidenced by MI-based BCI (Savic et al., 2021). Patients in the intermediate to advanced stages of ALS, exhibiting significantly restricted motor function, may derive advantages from semi-invasive (e.g., ECoG-based BCI; Pels et al., 2019; Wyse-Sookoo et al., 2024) or invasive BCIs to acquire more steady and accurate neural signals, facilitating more sophisticated and efficient communication control. These devices can markedly improve patients’ communicative efficiency and augment their ability for social interaction. Nonetheless, invasive and semi-invasive BCIs, albeit providing superior performance, entail increased surgical risks and long-term safety issues. As a result, in clinical settings, numerous individuals with moderate to severe disabilities persist in choosing non-invasive BCIs (e.g., P300-based BCI; Wolpaw et al., 2018) or fNIRS-based BCI (Borgheai et al., 2020). Despite exhibiting marginally reduced signal stability, these systems have a robust safety profile and facilitate user-friendly operation, thereby delivering efficient communication support and markedly enhancing patients’ quality of life.

### 9.2 BCI integrates cutting-edge technologies: a new pathway for ALS intervention

Virtual reality (VR) is a digital technology that creates immersive virtual environments via computer systems, employing multi-source information fusion and interactive simulation techniques to deliver multi-sensory experiences, encompassing visual and audio simulations. Although there is no direct scientific evidence regarding the use of BCI-VR fusion technology for ALS patients, studies have integrated the two for stroke patients, producing the following outcomes: Research demonstrates that the average recognition rate in the VR scene group attained 74.6% post-training, markedly surpassing the 67.3% in the static scene group and the 70.5% in the dynamic scene group. This further illustrates that VR environments augment the recognition efficacy of subjects’ EEG signals (Xie et al., 2022). Immersive experiences can diminish external distractions and enhance patient training results. The strategic focus on advancing medical applications of BCIs, along with ongoing technological advancements in flexible electrodes and hybrid decoding algorithms, will facilitate the transition of BCI and VR from theoretical frameworks to clinical validation. This convergence offers potential in overcoming the shortcomings of conventional assistive technologies—such as tedious interaction and inadequate adaptability—providing ALS patients with more human-centred and effective therapeutic and helpful solutions.

Neurophysiological investigations in ALS patients indicate that heightened excitability in the motor cortex may contribute to neurodegeneration. Repetitive transcranial magnetic stimulation (rTMS) might diminish motor cortical excitability via low-frequency stimulation (e.g., 1 Hz) or particular stimulation protocols (e.g., continuous theta burst stimulation, cTBS), consequently mitigating glutamatergic excitotoxicity and decelerating illness progression (Jiménez-García et al., 2024). There are studies (Dileone et al., 2010; Di Lazzaro et al., 2014) examined the impact of continuous cTBS on ALS, including a preliminary randomised trial with 20 ALS patients in which stimulation was administered to the primary motor cortex (M1). Results indicated a deceleration in disease progression following 6 months of treatment. Numerous minor trials have evaluated diverse stimulation treatments to mitigate excitotoxicity in ALS. These studies collectively indicate that neuromodulation may provide moderate benefits in decelerating disease development, while the existing evidence is still preliminary. Although extensive clinical trials have not directly integrated rTMS with BCI technology in ALS patients, pertinent research and technical advancements offer theoretical underpinnings and practical viability for this amalgamation. Future investigations may delve into particular uses of integrated rTMS and BCI technology, including the creation of adaptive rTMS systems informed by BCI feedback to attain enhanced neuromodulation precision.

Deep brain stimulation (DBS) is a neurostimulation method that modulates aberrant neural activity through the implantation of electrodes in targeted deep brain areas to provide electrical stimulation (Krauss et al., 2021). Presently, DBS has emerged as a principal therapy modality for managing mid-to-late stage Parkinson’s disease. According to research by Passera et al. (2023), DBS markedly enhances motor symptoms in PD patients, reducing tremors and rigidity. The mean reaction time decreases from roughly 600 milliseconds with DBS deactivated to approximately 400 milliseconds with DBS activated, thereby improving patients’ capacity to engage in daily activities. Furthermore, DBS can influence neuronal activity in the motor cortex (Soreq et al., 2013), therefore enhancing patients’ motor function and overall quality of life. ALS, a neurodegenerative condition, may gain new prospects through the future amalgamation of BCI and DBS technologies, facilitating patient interaction with their surroundings. DBS technology modulates brain activity via electrical stimulation to enhance neurological function. Integrating these methodologies offers potential for delivering more accurate neural modulation to ALS patients, improving motor function and communication skills, therefore raising their quality of life. However, existing studies with small sample sizes, low doses, and sensitive endpoints are not yet important to support it as a standard treatment (Ranieri et al., 2020).

Functional Electrical Stimulation (FES) is a neuromodulation method that employs electrical pulses with defined parameters to stimulate injured nerves or muscles, thereby activating neural pathways to restore or compensate for motor or sensory capabilities. It is extensively utilised in the rehabilitation of neurological illnesses (Atkins and Bickel, 2021). FES theoretically possesses potential in activating paralysed muscle units in late-stage ALS patients and postponing the advancement of muscle atrophy. Current study substantiates that the integration of FES with BCI significantly enhances motor function rehabilitation in individuals with neurological impairments, including stroke or spinal cord injury (Biasiucci et al., 2018). Nonetheless, the clinical efficacy of FES in ALS remains inadequately substantiated by extensive investigations. Research by Herrmann et al. indicates that pharyngeal electrical stimulation (PES) may not enhance swallowing performance in ALS patients, although this single-centre, small-sample exploratory study has inherent limitations. Conversely, the fundamental mechanism via which FES influences functional status in ALS patients remains ambiguous. In conclusion, there is an urgent necessity for additional high-quality, large-scale systematic investigations to clarify the application value and mechanism of action of FES in ALS. Nonetheless, owing to the progressive characteristics of ALS, people demonstrate variable levels of muscular denervation. Furthermore, certain research indicates that FES may not produce beneficial outcomes for ALS patients (Herrmann et al., 2022). This presents difficulties for the implementation of FES-BCI in rehabilitating motor function for ALS patients, requiring additional research.

## 10 Future prospects and limitations of BCIs in ALS

This work succinctly delineates the core ideas and principles of numerous BCI paradigms, comprehensively summarising their application principles and present developmental state in the context of ALS. Experts are increasingly concentrating on ALS sufferers, striving to utilise BCIs to aid or substitute their speech and movement skills through the integration of varied technology. Statistics reveal that ALS patients possess elevated expectations for BCIs, especially concerning communication-related alert functionalities. They expect to attain a level of self-sufficiency with BCI technology (Kageyama et al., 2020; Nakamura et al., 2024). Future multidisciplinary collaboration will enhance research in the BCI domain, facilitating more effective applications in ALS treatment.

Nonetheless, BCI systems utilised for ALS sufferers exhibit numerous deficiencies that necessitate additional enhancements and optimisation, which may also indicate prospective avenues for BCI advancement: (1) although the stability of current BCIs is guaranteed, they are limited to decoding just brief utterances. Effectively capturing patient intent in real-time within intricate situations continues to pose a considerable problem; (2) the existing BCI frameworks for ALS patients are comparatively restricted and fail to address the varied requirements of each individual. Future progress must prioritise the establishment of diverse paradigms to meet the distinct needs of each ALS patient.

Current developments in BCI research are swiftly progressing in various directions, including both hardware and software components. The advancement of high-density electrode arrays signifies a notable trend. Neuralink’s flexible electrode array, like a wire, is engineered for minimally invasive brain implantation, minimising tissue injury while enhancing the quantity and quality of signal acquisition channels (Musk, 2019). In hardware, machine learning and artificial intelligence technologies are progressively utilised in signal decoding. Advanced neural network models are extensively utilised to interpret motor intentions, voice data, and cognitive states from high-dimensional intrusive brain input (Robitaille et al., 2023).

Nonetheless, BCI technology continues to encounter obstacles in specific domains. Non-invasive BCIs necessitate advancements in the precision of brain signal capture, improved spatial resolution, and the mitigation of motion artefacts and external noise. Invasive BCIs must immediately resolve biocompatibility concerns linked to prolonged implantation, since tissue encapsulation of electrodes leads to substantial signal loss within roughly 6 months (Colucci et al., 2022). Advancements are still required in the fundamental chips and algorithms of BCI technology. At present, there exists a deficiency of specialised chips for the capture and processing of brain signals. To attain high-channel, high-speed real-time brain-computer communication, it is essential to build analog-to-digital conversion chips and wireless transmission chips tailored for neural signals, alongside enhancing decoding efficiency via algorithmic optimisation (Guo et al., 2024). Moreover, universality and individual diversity provide considerable obstacles. Due to the variability of brain signals among persons and over time, maintaining the stability of BCI systems across diverse users and days necessitates adaptive learning algorithms for ongoing calibration and modification (Kim et al., 2024).

As BCI technology becomes more integrated into everyday life, it presents a range of unexpected ethical and legal dilemmas. The major concern is neuronal privacy. By interpreting brain impulses via BCIs, it becomes feasible to mistakenly “glimpse” into users’ thoughts and mental states. The potential damage from the abuse or leakage of such data cannot be overstated. Consequently, there is an imperative necessity to explicitly delineate ownership and usage restrictions for brain data to avert the possible exploitation of this “mind-reading” capability (Yang and Jiang, 2025).
